# Defense Priming in *Nicotiana tabacum* Accelerates and Amplifies ‘New’ C/N Fluxes in Key Amino Acid Biosynthetic Pathways

**DOI:** 10.3390/plants9070851

**Published:** 2020-07-06

**Authors:** Nils Hanik, Marcel Best, Michael J. Schueller, Ryan Tappero, Richard A. Ferrieri

**Affiliations:** 1 Fachbereich Chemie, Johannes Gutenberg Universität, 55099 Mainz, Germany; nils.hanik@hevs.ch (N.H.); best@uni-heidelberg.de (M.B.); 2 Missouri Research Reactor Center, University of Missouri, Columbia, MO 65211, USA; schuellerm@missouri.edu; 3 Chemistry Department, University of Missouri, Columbia, MO 65211, USA; 4 Brookhaven National Laboratory, National Synchrotron Light Source Division, Upton, NY 11973, USA; rtappero@bnl.gov; 5 Division of Plant Sciences, Interdisciplinary Plant Group, University of Missouri, Columbia, MO 65211, USA

**Keywords:** isotope ratio analysis, carbon-11, nitrogen-13, amino acid metabolism, X-ray fluorescence imaging, defense priming, plant insect herbivory

## Abstract

In the struggle to survive herbivory by leaf-feeding insects, plants employ multiple strategies to defend themselves. One mechanism by which plants increase resistance is by intensifying their responsiveness in the production of certain defense agents to create a rapid response. Known as defense priming, this action can accelerate and amplify responses of metabolic pathways, providing plants with long-lasting resistance, especially when faced with waves of attack. In the work presented, short-lived radiotracers of carbon administered as ^11^CO_2_ and nitrogen administered as ^13^NH_3_ were applied in *Nicotiana tabacum*, to examine the temporal changes in ‘new’ C/N utilization in the biosynthesis of key amino acids (AAs). Responses were induced by using topical application of the defense hormone jasmonic acid (JA). After a single treatment, metabolic partitioning of recently fixed carbon (designated ‘new’ carbon and reflected as ^11^C) increased through the shikimate pathway, giving rise to tyrosine, phenylalanine and tryptophan. Amplification in ‘new’ carbon fluxes preceded changes in the endogenous (^12^C) pools of these AAs. Testing after serial JA treatments revealed that fluxes of ‘new’ carbon were accelerated, amplified and sustained over time at this higher rate, suggesting a priming effect. Similar results were observed with recently assimilated nitrogen (designated ‘new’ nitrogen reflected as ^13^N) with its partitioning into serine, glycine and glutamine, which play important roles supporting the shikimate pathway and downstream secondary metabolism. Finally, X-ray fluorescence imaging revealed that levels of the element Mn, an important co-factor for enzyme regulation in the shikimate pathway, increased within JA treated tissues, suggesting a link between plant metal ion regulation and C/N metabolic priming.

## 1. Introduction

Herbivory by leaf-feeding insects often occurs through a rapid onset of attack, which, if left unchecked, can result in extensive loss of photosynthetic tissue, diminishing input of essential resources in support of growth. Many plants have evolved with sophisticated defense mechanisms that enable them to respond physiologically and metabolically to insect attack, but these actions often come at a high energy cost [1,2,3,4]. Some plant defenses are inducible, enabling plants to avoid these high energy costs in the absence of attack. Even so, the ability of the plant to mount any defense in response to herbivore attack takes time. In a coordinated timeline, early events can span milliseconds to minutes of initial damage and can involve perturbations to the plasma membrane potential, variation in cytosolic Ca^+2^ levels and variation in levels of reactive oxygen species [5]. These events allow for early recognition of attack, but more importantly provide a gauge for assessing the severity of damage caused by feeding, which can trigger downstream response networks involving protein kinases [6] and release of phytohormones [7] such as jasmonic acid (JA), its methyl ester (MeJA) and JA-conjugates promoting plant-wide signal transduction [8].

There can also be tradeoffs to mounting a defense against different attacking species. Defending against one attacker could increase susceptibility to another. Spatial and temporal variability that defines the severity of attack by one species must be considered [9].

While inducible defenses enable plants to avoid the high cost of implementing a chemical defense response in the absence of attackers, the downside of this is plants could suffer considerable damage in the time it takes for such a defense response to be mounted. To adjust for this vulnerability many plants have been shown to possess a highly adaptive trait known as defense priming [10], where the defense response network can be deployed in a faster, stronger and/or sustained manner following the perception of signaling cues, such those as described above, involving JAs or other chemical compounds [11,12]. Priming has its underpinnings at the molecular level, where changes in chromatin and/or the accumulation of mRNA of genes associated with signaling roles in plant defense, as well as changes in signaling proteins and pattern-recognition receptors and/or metabolites, can support faster and stronger responses when triggered by certain signals [13,14,15].

Signaling substances such as JAs belong to a ubiquitous class of phytohormones that is found in all higher plants. Phytohormones fulfill many roles in plant growth, promoting physiological processes [16,17,18] and stimulating many natural developmental processes, including embryogenesis, seed germination, flowering, pollen production [17,19,20] and accumulation of storage proteins [21] during late-season senescence [22]. However, JAs are best known for their role in plant defense as a response signal to tissue damage, pathogen infection and herbivory [23,24,25,26,27], cuing attack at both local and systemic tissue levels [8,28,29,30]. JAs are known to promote transcription of numerous genes [31] that impact whole-plant resource allocation of nitrogen- and carbon-containing substances [26,32,33] which can trigger metabolic responses at cellular levels [17,34,35,36,37,38,39,40]. Such responses are typically coordinated with the upregulation of specialized biochemical pathways that are important to the plant’s overall defense response [39,41,42]. For example, phenolic acid metabolism in plants requires the initial steps of general phenylpropanoid metabolism providing precursors for the synthesis of lignin, tannins, flavonoids and other phenolics that serve to build physical barriers and/or reserves of protective allelochemicals in plant defense [24]. It is well-known that JAs will induce transcription of key enzymes, including phenylalanine ammonia lyase [43,44] and caffeoyl CoA O-methyltransferase [45], that promote the deamination of phenylalanine, the primary substrate of this pathway, as well as catalyze the O-methylation of key intermediates, leading to increased lignification [43,45,46].

**Figure 1:**
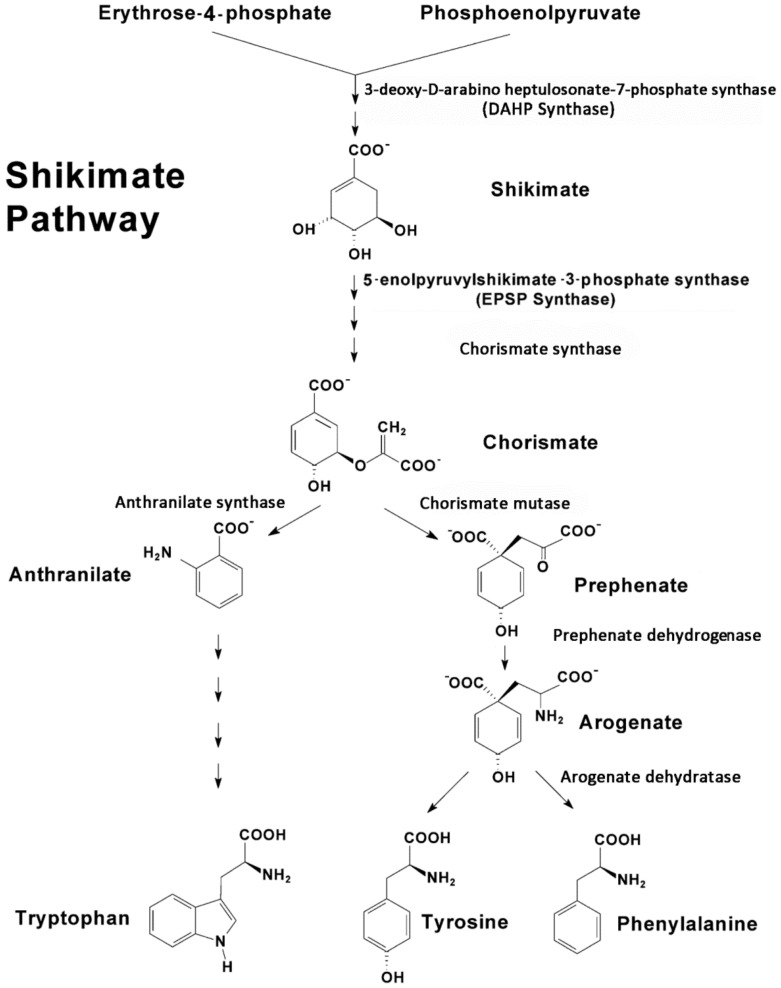
The shikimate pathway is comprised of several pathways that are controlled by several regulatory enzymes, including 3-deoxy-D-arabino-heptulosonate 7-phosphate synthase, chorismate synthase, chorismate mutase, prephenate dehydrogenase, arogenate dehydratase and anthranilate synthase, to name a few. Tyrosine and phenylalanine share a common branching pathway from chorismate through arogenate. Tryptophan derives from a separate branching pathway from chorismate through anthranilate.

Upstream, the shikimate pathway (Figure 1) is responsible for the biosynthesis of key aromatic compounds providing necessary building blocks in support of downstream secondary defense chemistry. In addition to the noted role of phenylalanine in plant defense, tyrosine and tryptophan also play important roles here [47,48]. Hence, the shikimate pathway is seen as an important gateway to plant defense chemistry.

The shikimate pathway proceeds through several enzyme-driven steps to a branch point intermediate chorismate [49] that is eventually converted to these three aromatic amino acids (AAs). The entire process is coupled tightly via metabolite feedback control mechanisms impacting several regulatory enzymes in the pathway, including 3-deoxy-D-arabino-heptulosonate 7-phosphate synthase (DAHP synthase), chorismate synthase, chorismate mutase, prephenate dehydrogenase, arogenate dehydratase and anthranilate synthase, to name a few [50,51].

In a past publication, Schwachtje and Baldwin [52] suggested that a key link in the plant defense network is reprogramming of primary metabolic pathways, such as this, where aromatic AAs not only serve as the chemical building blocks for downstream chemistry, but may themselves serve as signals that activate these downstream pathways. However, to the best of our knowledge, little is known about the temporal regulation of the shikimate pathway as part of plant’s primary metabolic landscape and most particularly how this pathway may be affected in the context of plant defense priming.

Here, we sought to provide evidence of metabolic defense priming, using radioactive isotopes of carbon (^11^C, t_½_ 20.4 min) and nitrogen (^13^N, t_½_ 9.97 min) coupled with metabolite isotope ratio analyses, to show that the shikimate pathway can be reprogrammed in response to JA treatment, where its state remains activated over time. The work presented was conducted in *N. tabacum*, which was chosen as a model system for examining plant defenses, as it has a rich alkaloid defense biochemistry that is responsive to JA based on our prior work [8,53].

## 2. Results and Discussion

**Figure 2:**
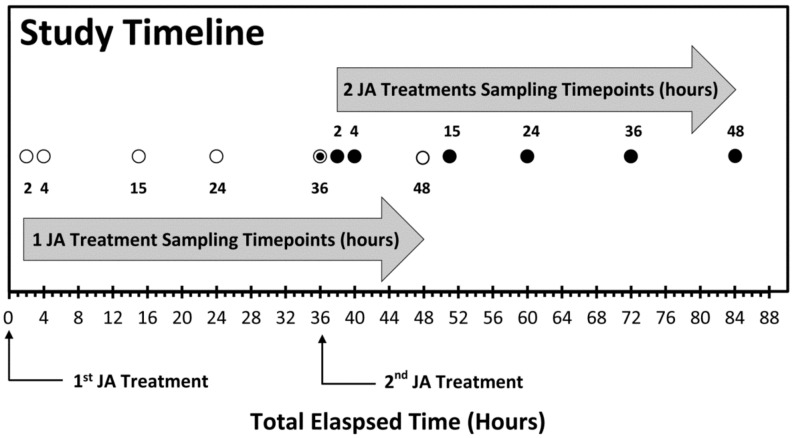
Study timeline showing sampling timepoints after one jasmonic acid (JA) treatment and two JA treatments.

In this work, we examined the effects of sequential JA treatments on the temporal profile for metabolic partitioning of newly fixed carbon (as ^11^C) and newly assimilated nitrogen (as ^13^N) into key metabolite pools relative to the changes seen in the endogenous levels of metabolites within these pools. In the study design, local tissue responses were first examined for the effects of treatments on the metabolic partitioning of newly fixed carbon into [^11^C]sugar and [^11^C]amino acid pools over timepoints of 2, 4, 15, 24, 36 and 48 h post-JA treatment (Figure 2). Next, local and systemic tissues were further tested for changes in individual metabolites, at timepoints of 2, 4, 15, 24 and 36 h post-JA treatment. Local tissue was defined as source leaf-3 (the third fully expanded leaf, counting down from the top of the plant). Systemic tissues that were tested included the young developing leaves of the plant’s apex. The process was extended to include testing at the same timepoints after a second JA treatment was applied 36 h after the first JA treatment. Separate age-matched plants were used for each sampled timepoint, totaling 30–50 plants for the entire study series. Additionally, baseline control responses for comparing effects of one JA treatment were measured at timepoint zero in the Figure 2 timeline, while a second set of baseline responses for comparing effects of two JA treatments were measured at 36 h into the time series, to eliminate confounding effects of plant growth.

**Figure 3:**
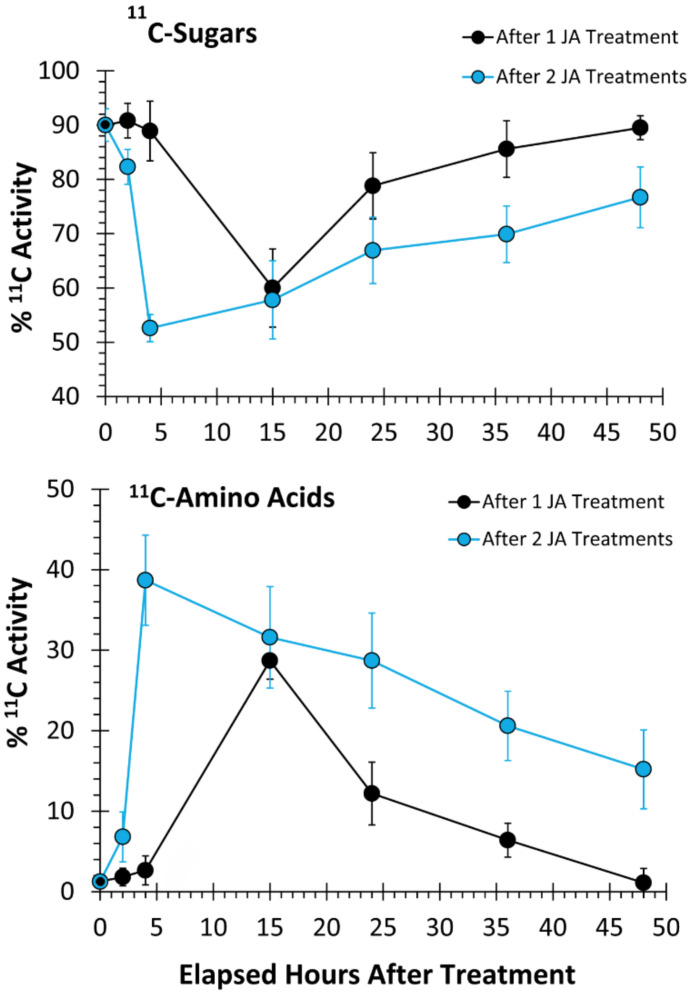
Local tissue responses to 500 µM JA treatments—metabolic partitioning of ‘new’ carbon resources (as ^11^C) into soluble sugar and amino acid pools. Data are presented as relative % ^11^C activity from load leaf solvent extraction and reflect N = 3 5 biological replicates ± SE.

In unstressed plants, source leaf-3 partitioned 90 ± 3% of its ‘new’ carbon (as ^11^C) into soluble [^11^C]sugar pools (Figure 3) with >95% of this fraction comprised largely of [^11^C]sucrose, while only 2 ± 1% of this carbon source was partitioned into [^11^C]AAs. Mock treatments using deionized water did not induce a metabolic response of ‘new’ carbon partitioning into these pools when tested at 4 and 15 h post-treatment.

A single treatment using 500 µM JA did not show an induced metabolic response at 2 and 4 h post-treatment (Figure 3, one JA Treatment). However, at 15 h post-treatment, ‘new’ carbon partitioning into soluble sugars decreased significantly to 60 ± 7% balanced by a significant increase in ‘new’ carbon partitioning into AAs to 30 ± 8% consistent with our earlier findings, using MeJA treatments [53]. In that earlier study, we did not perform mock treatments to disentangle a wounding response from a JA signaling response. Our rapid ‘patching’ of the administration sites, using lanolin paste, appeared to minimize the effects of mechanical wounding, at least in our examination of metabolic regulation. Likely, these effects were well below those of JA treatment such that the mock treatments did not show a response. Furthermore, we extended testing of metabolic responses in the present work to include additional timepoints beyond those of the original work. By extending the temporal profile to later timepoints, we were able to observe ‘new’ carbon partitioning, eventually returning to a normal unstressed state by 36 to 48 h post-treatment. Using this knowledge, we looked for evidence of defense priming by administering a second JA-treatment 36 h after the first JA-treatment. When two JA treatments were applied in sequence, using a 36-hour protocol (Figure 2), the change in ‘new’ carbon metabolic partitioning in sugar and AA pools between one JA treatment and two JA treatments was accelerated after the second treatment showing its greatest change now at 4 h post-treatment (Figure 3). Here, partitioning of ‘new’ carbon away from the soluble sugar pool was even greater decreasing significantly to 52 ± 4% and balanced by an even greater increase in ‘new’ carbon partitioning into AAs to 38 ± 5%. Furthermore, ‘new’ carbon fluxes remained elevated at the 48-hour timepoint.

**Figure 4:**
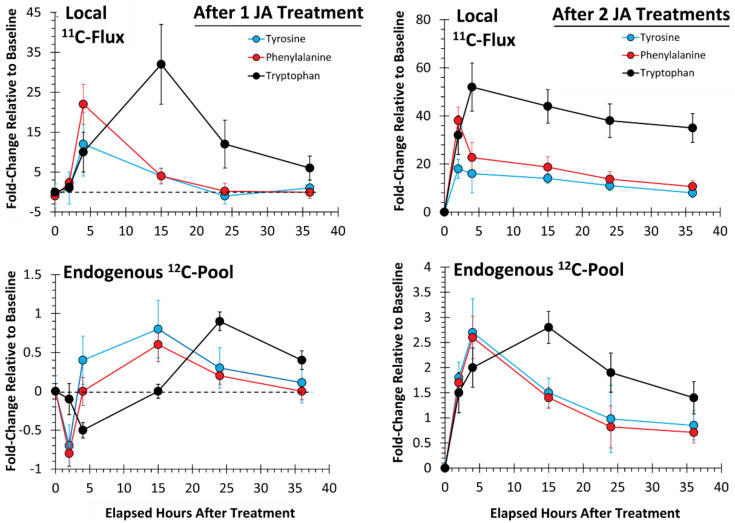
Local tissue responses to 500 µM JA treatments—partitioning of ‘new’ carbon (as ^11^C) into tyrosine, phenylalanine and tryptophan is presented after a single mock treatment and after sequential JA treatments (top panels). Change in the endogenous levels (^12^C) of these amino acids (AAs) is presented in the bottom panels. Data (N = 3) reflect the fold-change relative to baseline ± SE at timepoints 2, 4, 15, 24 and 36 h post-treatment.

As an extension of our earlier work [53], we examined the temporal profile for metabolic partitioning of ^11/12^C sources into aromatic AAs, tyrosine, phenylalanine and tryptophan derived from the shikimate pathway (Figure 4). Mock treatment tested at 2 and 4 h post-treatment did not result in a change in carbon resource partitioning, again suggesting that the study series reflected responses to JA-treatment and not mechanical wounding. However, with one JA treatment, ‘new’ carbon partitioning (as ^11^C) into tyrosine and phenylalanine (Figure 4, upper left-side panel) increased significantly, peaking at 4 h post-treatment, at 12 ± 6 (*p* = 0.042) and 22 ± 5 (*p* = 0.015) fold-change relative to baseline, respectively, and returning to unstressed baseline levels by 24 h post-treatment. Partitioning of ‘new’ carbon into tryptophan also increased significantly after one JA treatment, peaking at 15 h post-treatment, at 32 ± 9 fold-change (*p* = 0.039) relative to baseline before returning to an unstressed baseline level by 36 h post-treatment. Different response times for tyrosine and phenylalanine versus tryptophan point to different branch point kinetics at the chorismate intermediate (Figure 1) and passing through prephenate/arogenate versus anthranilate in route to these end-products.

Behavior in the change in ^12^C endogenous levels of these AAs after one JA treatment exhibited a different trend (Figure 4, lower left-side panel). However, tyrosine and phenylalanine levels exhibited a sinusoidal behavior with one JA treatment, initially decreasing significantly to their lowest levels at 2 h post-treatment to −0.7 ± 0.3 (*p* = 0.042) and −0.8 ± 0.2 (*p* = 0.039) fold-changes, respectively, relative to baseline, and then elevating significantly to 0.8 ± 0.4 (*p* = 0.022) and 0.6 ± 0.3 (*p* = 0.041), respectively, at 15 h post-treatment, before returning to unstressed baseline levels at 36 h post-treatment. Response of tryptophan was similar, though delayed in time, much like the ^11^C data presented for ‘new’ carbon flux. Endogenous tryptophan levels initially declined significantly to −0.5 ± 0.1 fold-change (*p* = 0.018) relative to baseline, at 4 h post-treatment, and then they rose significantly, to a maximum 1.0 ± 0.1 fold-change (*p* = 0.012), at 24 h post-treatment, before declining again, not quite to baseline, to a level of 0.4 ± 0.1 fold-change, at 36 h.

Taken together, we believe the initial decline in the endogenous ^12^C aromatic AA pools was the result of these pools being depleted in support of downstream secondary metabolism. As the shikimate pathway is highly nested with feedback loops of regulation [54], this depletion likely triggered upregulation of the shikimate pathway which manifested 2 h later in the upregulation of ‘new’ carbon fluxes. However, as the endogenous ^12^C AA pools were replenished and exceeded baseline levels by 15 h post-treatment, the metabolic machinery underpinning the shikimate pathway was downregulated, as reflected by the decreased fluxes of ^11^C.

Evidence of defense priming, however, becomes apparent at the local tissue level, both in the level of change observed for metabolic turnover and in the response times when tissues were exposed to two JA treatments (Figure 4, upper right-side panel). The change in ‘new’ carbon partitioning (as ^11^C) into tyrosine and phenylalanine was significantly elevated to a 19 ± 2 (*p* = 0.017) and 39 ± 5 (*p* = 0.009) fold-change, respectively, relative to baseline levels by 2 h post-treatment. These levels of change reflected amplifications in metabolic turnover of 1.6× (*p* = 0.059) and 1.8× (*p* = 0.062), respectively, relative to the observed change after one JA treatment. Though not statistically significant, the trends in metabolic amplification over time seem clear. Furthermore, the response time was halved after two JA treatments, indicating acceleration in metabolic turnover. As before, the response time reflecting changes in ^11^C partitioning into tryptophan lagged slightly behind those of tyrosine and phenylalanine, peaking now at 4 h post-treatment, at 55 ± 8 fold-change relative to baseline, reflecting a significant 1.7× amplification (*p* = 0.046) in turnover, and a quartering in the response time.

Additionally, tyrosine and phenylalanine endogenous pools (Figure 4, lower right-side panel) no longer exhibited sinusoidal behavior as with one JA treatment, but rather rose rapidly, peaking significantly now at 4 h post-treatment, to levels of 2.7 ± 0.4 (*p* = 0.014) and 2.6 ± 0.3 (*p* = 0.029) fold-change, respectively, after two JA treatments. These levels of change reflected 4.3× amplifications in metabolic turnover for both AAs that was significant (*p* = 0.042 and *p* = 0.039, respectively) and a quartering in the response time. Furthermore, both AA levels remained elevated above baseline, at 36 h post treatment. Like before, the response of the endogenous tryptophan pool exhibited a delayed response relative to tyrosine and phenylalanine, peaking significantly at 2.7 ± 0.2 (*p* = 0.004) fold-change relative to baseline, at 15 h post-treatment, reflecting a 3.4× amplification in turnover that was significant (*p* = 0.036), and slightly less than halving in response time. Tryptophan levels also remained elevated above baseline at 36 h post-treatment.

**Figure 5:**
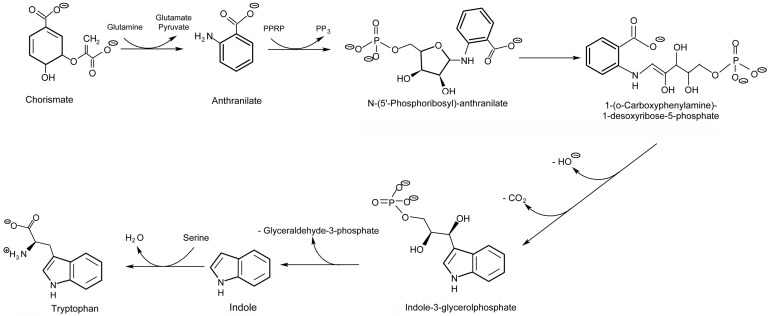
Involvement of glutamine and serine in the shikimate pathway.

Follow-up studies using the same protocol with the ^13^NH_3_ tracer allowed us to identify glutamine, serine and glycine as responsive to JA treatments, where the metabolic turnover of newly assimilated nitrogen (as ^13^N) into these metabolites showed similar elevations and temporal profile to that seen with the aromatic AAs. Amongst its many roles in primary metabolism, glutamine is also important to the shikimate pathway, as it provides a source of N for amination of chorismate producing anthranilate (Figure 5). Serine is also involved in support of this pathway, as it couples with indole to produce tryptophan. Furthermore, serine and glycine are important in one-carbon metabolism and are critical to the support of downstream secondary defense chemistry in the phenylpropanoid pathway [55].

**Figure 6:**
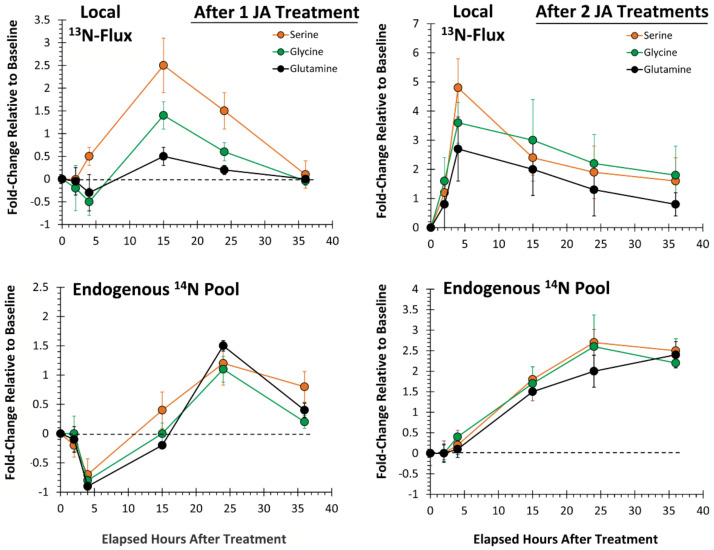
Local tissue responses to 500 µM JA treatments—partitioning of ‘new’ nitrogen (as ^13^N) into serine, glycine and glutamine is presented after sequential JA treatments (top panels). Change in the endogenous levels (^14^N) of these AAs is presented in the bottom panels. Data (N = 3) reflect the fold-change relative to baseline ± SE at timepoints 2, 4, 15, 24 and 36 h post-treatment.

Here, we examined the temporal profile for metabolic partitioning of ^13/14^N sources into serine, glycine and glutamine (Figure 6). After one JA treatment, ‘new’ nitrogen partitioning (as ^13^N) into all three AAs (Figure 6, upper left-side panel) increased significantly, peaking at 15 h post-treatment, at levels of 2.5 ± 0.6 (*p* = 0.028), 1.5 ± 0.2 (*p* = 0.036) and 0.6 ± 0.1 (*p* = 0.049) fold-change relative to baseline, respectively, and returning to unstressed baseline levels by 36 h post-treatment. After two JA treatments (Figure 6, upper right-side panel), partitioning of newly assimilated nitrogen increased significantly relative to baseline, peaking now at 4 h post-treatment, at levels of 4.9 ± 0.8 (*p* = 0.029), 3.6 ± 0.5 (*p* = 0.044) and 2.8 ± 0.3 (*p* = 0.047) fold-change, respectively. These changes reflected amplifications in metabolic turnover of 2× (*p* = 0.050), 2.4× (*p* = 0.031) and 4.7× (*p* = 0.050), respectively, over that observed from one JA treatment, and a quartering of the response time.

Similar to behavior of the aromatic AAs, change in endogenous levels of serine, glycine and glutamine after one JA treatment (Figure 6, lower left-side panel) exhibited a sinusoidal behavior with significant decreases to minimum levels of −0.7 ± 0.2 (*p* = 0.033), −0.8 ± 0.2 (*p* = 0.039) and −0.9 ± 0.1 (*p* = 0.027), respectively, relative to baseline occurring at 4 h post-treatment, then significant increases to maximum levels of 1.2 ± 0.2 (*p* = 0.037), 1.5 ± 0.2 (*p* = 0.031) and 0.9 ± 0.1 (p = 0.046), respectively, relative to baseline occurring at 15 h post-treatment. After two JA treatments, the sinusoidal behavior disappeared. Metabolic turnover of all three AAs increased nonlinearly from baseline, remaining elevated at 36 h post-treatment.

**Figure 7:**
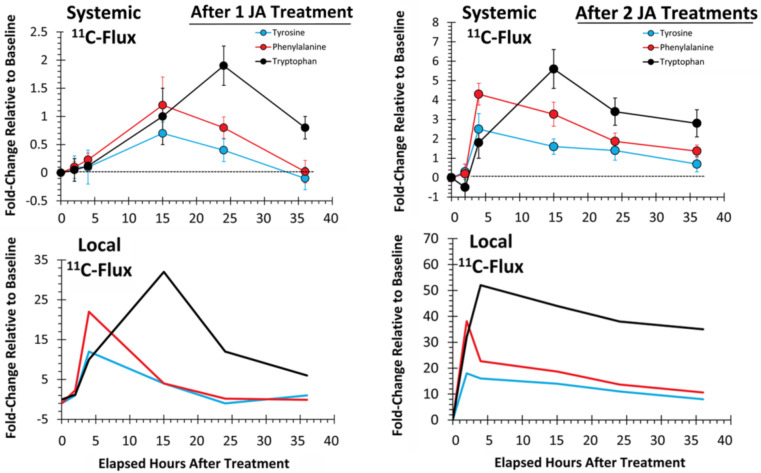
Systemic tissue responses to 500 µM JA treatments—partitioning of ‘new’ carbon (as ^11^C) into tyrosine, phenylalanine and tryptophan aromatic AAs is presented after sequential JA treatments (top panels). Data (N = 3) reflect the fold-change relative to baseline ± SE at timepoints 2, 4, 15, 24 and 36 h post-treatment. The ^11^C temporal profiles for local tissue responses from Figure 4 are shown in the lower panels for a comparison.

We also considered whether there was evidence of defense priming in systemic tissues (Figure 7). Here, we examined changes in ‘new’ carbon partitioning (as ^11^C) into tyrosine, phenylalanine and tryptophan. As seen in Figure 4, we observed similar temporal behavior for the metabolic turnover of these AAs after one JA treatment where tyrosine and phenylalanine exhibited a different profile than tryptophan. Here, tyrosine and phenylalanine peaked in turnover at 15 h post-treatment, while tryptophan peaked at 24 h post-treatment. We note that there was a consistent lag in the metabolic responses for systemic tissues when comparing Figure 7 upper (representing systemic tissue response times) to the lower panels (representing the local tissue response times from Figure 4). We attribute this lag in response to the time needed for transference of the JA signal from the local tissue, where it was applied to the systemic tissues, where we were testing metabolic response. From our past work using [^11^C]MeJA [8], we determined that such transport of signal, measured at a rate of transport of 10.8 ± 1.2 mm min^−1^, would take 2–3 h to reach these distal tissues. More importantly, the behavior that we observed after one JA treatment was amplified, accelerated and sustained at a higher turnover rate after two JA treatments, supporting the theory that defense priming is a plant-wide action.

**Figure 8:**
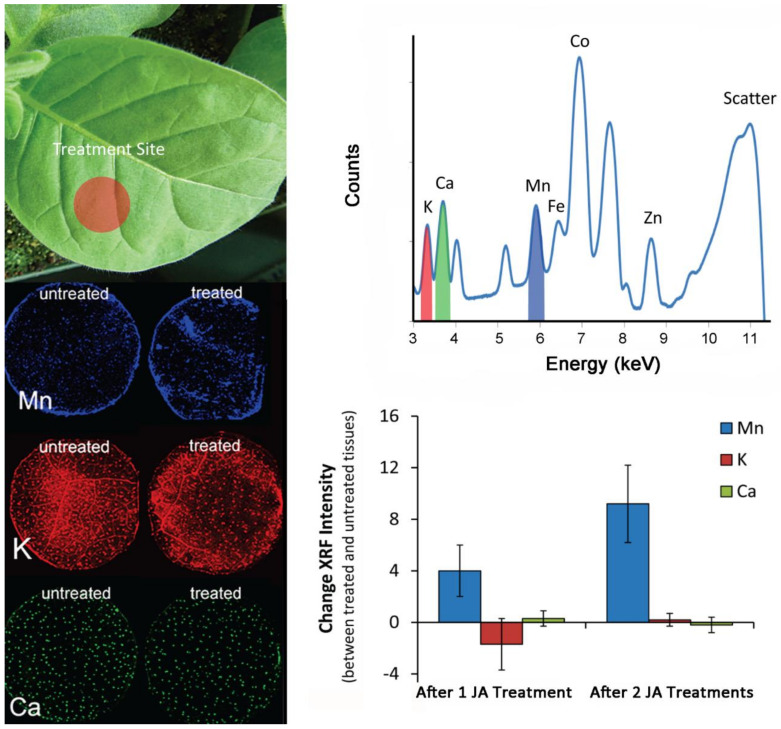
Synchrotron-based X-ray fluorescence imaging allows for spatial mapping of metals in leaf tissue based on their characteristic fluorescence signature after having absorbed the energy from a high-energy X-ray source. Metals will absorb X-rays and fluoresce at different energies characteristic of the element and its orbital makeup of electrons (upper right-side panel). This feature enables the distinction of different metals in the same sample (left panel). Images were acquired 15 h after each JA treatment and comparisons made between tissues taken from treated and non-treated halves of the same leaf-3. Data (N = 3) were presented as the fold-change ± SE relative to non-treated tissue levels (lower right-side panel).

Finally, since metal ions are required in order for DAHP synthase to catalyze reactions within the shikimate pathway, we wanted to examine whether defense priming can be correlated with plant ion regulation. In particular, it has been shown that DAHP synthase requires Mn^+2^ as a bivalent metal ion cofactor in order for the enzyme to function properly [56]. Using synchrotron-based X-ray fluorescence imaging (XRF), we spatially mapped the distribution of manganese (Mn), along with two house-keeping metals, potassium (K) and calcium (Ca), to ensure that JA treatments did not influence leaf water conductance impacting metal ion trafficking (Figure 8). Images were acquired 15 h after each JA treatment.

XRF images acquired for K and Ca showed minor vascular tissue and leaf stomata (bright spots). The activity can be correlated with guard cells in the stomatal complex responsive to fluxes of K and Ca, enabling expansion and contraction and hence regulating the stomata function. Fluorescence intensities were integrated and tabulated in Figure 8 (lower right-side panel), showing that K and Ca levels were not affected by JA treatments. On the other hand, Mn levels increased after one JA treatment and amplified further after two JA treatments (*p* = 0.030). This observation suggests that a strong tie not only exists between plant C/N metabolism and metal homeostasis, but that the latter may be influenced by priming effects, as well.

## 3. Materials and Methods

### 3.1. Plant Growth

Tobacco plants (*Nicotiana tabacum* L. cv Samsun) were grown from seeds in ProMix potting mix, using 5-inch plastic pots that contained a slow-release fertilizer (14-14-14, Osmocote™ Smart-Release Plant Food Flower & Vegetable™, The Scotts Company, Marysville, OH). Pots were placed in a commercial growth chamber (Conviron, Inc., Winnipeg, Canada), where growth conditions were set to a 16-hour photoperiod, 350 μmol m^−2^ s^−1^ light intensity and temperatures of 24 °C/22 °C (light/dark), with humidity at 70–80%. Plants were grown to the V7 stage of development for all studies.

### 3.2. Production and Administration of Radioactive ^11^CO_2_

^11^CO_2_ (t_½_ 20.4 min) was produced on the BNL Ebco TR-19 Cyclotron (Ebco Industries Ltd., Richmond, BC, Canada), located at Brookhaven National Laboratory, using high-pressure research-grade N_2_ gas target irradiated with a 17 MeV proton beam to generate ^11^C via the ^14^N(p,α)^11^C nuclear transformation [57,58]. The ^11^CO_2_ was trapped on molecular sieve 4Å, desorbed and quickly released into an air stream at 400 mL min^−1^, as a discrete pulse for dosing a leaf affixed within a 5 × 10 cm lighted (350 µmol m^−2^ s^−1^) leaf cell, to ensure a steady level of fixation. The load leaf affixed within the cell was pulse-fed ^11^CO_2_ for 1 min and then chased with normal air, until tissues were harvested twenty minutes later.

### 3.3. Production of Radioactive ^13^NH_3_

^13^NH_3_ was produced via the ^16^O(p,n)^13^N nuclear transformation from triple distilled water, using a 2.5 mL volume high-pressure liquid target and 17 MeV protons from the TR-19 (Ebco Industries Ltd., Richmond, BC, Canada) cyclotron at Brookhaven National Laboratory. The ^13^N was recovered as carrier-free ^13^NO_3_^−^ and chemically reduced to ^13^NH_3_ gas, using DeVarda’s Alloy (Aldrich Chemical, St. Louis, MO, USA) [57]. Because the reduction of ^13^NO_3_^−^ to ^13^NH_3_ is a highly exothermic process, it was possible to administer the ^13^NH_3_ tracer as a discrete pulse that was released into an air stream, at 400 mL m^−1^, that fed the leaf cell. However, it should be noted that NH_3_ can be toxic to plants; therefore, the ^13^NH_3_ was generated at high specific activities that were in excess of 1 Ci µmol^−1^. That is, for approximately 20 mCi doses of tracer we typically administered to tissue, the plant received on the order of 20 nmol of ammonia exposure. Ammonia mass was measured by using Kitagawa gas-detection tubes (Kitagawa America, LLC., Pompton Lakes, NJ, USA). In a case study reported by Castro, Stulen and De Kok [59], using *Brassica olerace* L., neither manifested visible symptoms (such as black spots and necrosis) nor changes in tissue growth were observed with exposures below 4 mL L^−1^ of NH_3_ gas (or 235 µmol L^−1^). We estimate that our exposure levels were in the order of 10^2^-times less than this. Even so, we verified that leaf function was unaffected by tracer administration by measuring leaf photosynthesis during ^13^N administration, using infrared gas exchange (LI-6400, NB Li-Cor, Lincoln, NE, USA), and found no change.

**Figure 9:**
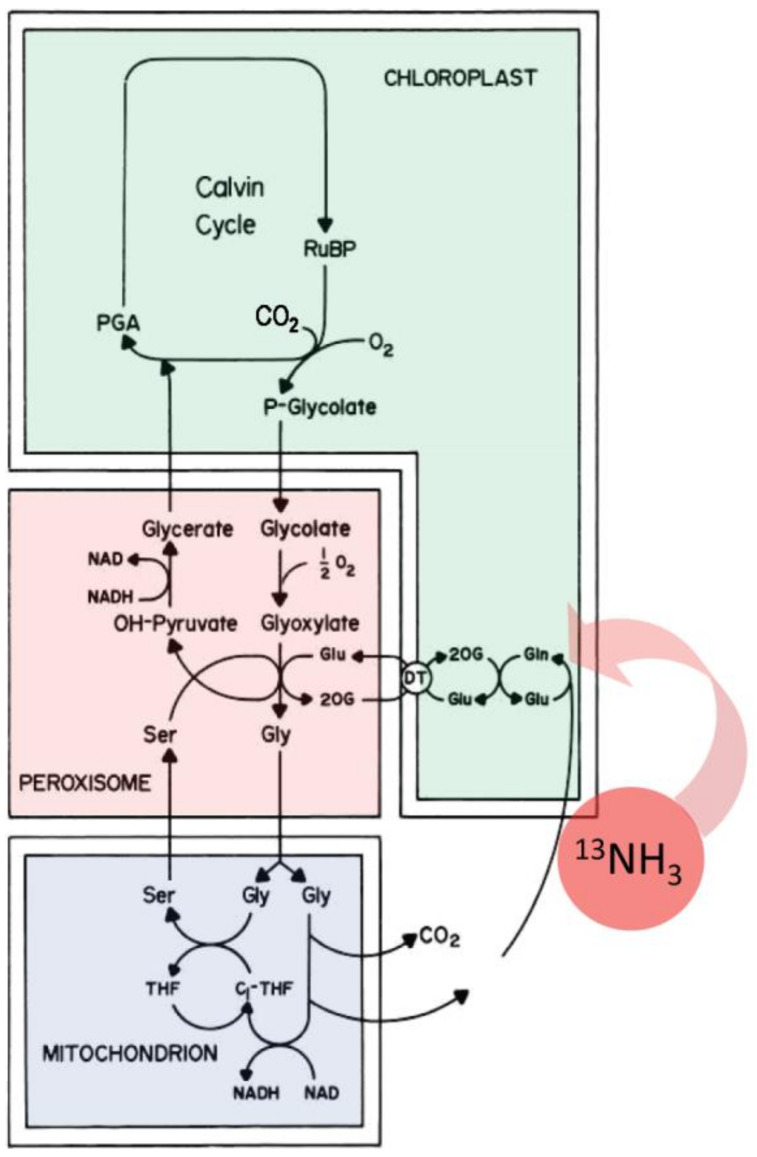
Assimilation of ^13^NH_3_ gas into the glutamine synthetase—glutamate synthase pathway provides a source of ‘new’ nitrogen in leaf amino acid biosynthesis.

Once administered, the radioactive ^13^NH_3_ gas was rapidly incorporated into the plant’s photorespiratory cycle [60,61] via the actions of the glutamine synthetase—glutamate synthase pathway (Figure 9) producing [^13^N]glutamine and [^13^N]glutamate. Through innercellular trafficking between the chloroplast, peroxisome and mitochondrion [62,63], other labeled AAs can be produced, providing a source of ‘new’ nitrogen within the leaf tissue.

### 3.4. JA Treatment and Radiotracer Administration

**Figure 10:**
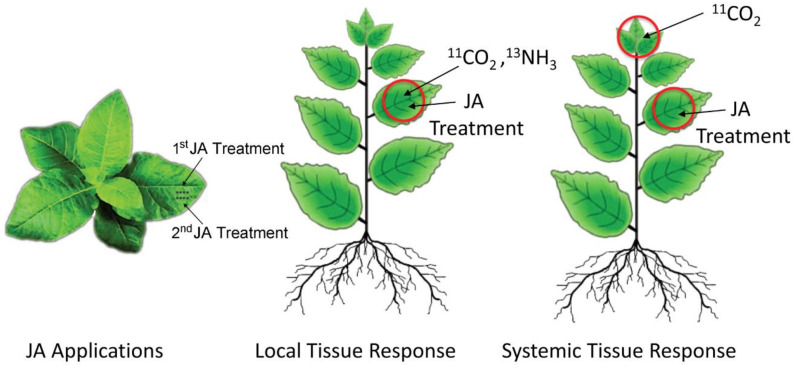
Visual protocol for testing local and systemic tissue responses to JA.

Pharmacological treatments of JA (500 µM) were administered by using a micro syringe to dispense JA to four tiny holes generated by using a fabric wheel applied to leaf-3 (Figure 10). Holes in the underside of the leaf were first sealed by using lanolin, and then after JA treatment, holes in the top of the leaf were sealed with lanolin. Mock treatments using de-ionized water were also applied for comparison. In the treatment series, either 1 or 2 JA treatments were applied, where, for a two-treatment study, the second treatment was applied 36 h after the first treatment. For local tissue response studies, we administered ^11^CO_2_ or ^13^NH_3_ to leaf-3 after the appropriate numbers of treatments were applied according to the timeline described above (Figure 2). Leaf designation was determined by counting down from the apex leaves, where leaf-1 was designated as the first leaf that was expanded to 50% of its full capacity. For systemic tissue response studies, we only tested using ^11^CO_2_ tracer administered to apex leaves after serial treatments of JA were applied to leaf-3.

### 3.5. Analysis of Plant Sugars

After ^11^CO_2_ pulsing and incubation for twenty minutes, exposed leaves were removed and subjected to metabolite analyses, following published procedures [64]. Tissues were flash-frozen in liquid nitrogen, ground to a fine powder and extracted in methanol:water (60:40 v/v) in Eppendorf™ tubes. After centrifugation, the insoluble fraction was separated from the liquid extract, and each was counted for ^11^C-activity, using a NaI gamma counter. The extract contained small water-soluble compounds, including sugars and amino acids. All data were decay corrected back to the end of bombardment, or end of cyclotron beam.

[^11^C]-Sugars were analyzed by radio thin layer chromatography (TLC), using glass-backed NH_2_-silica HPTLC-plates (200 µm, w/UV254) purchased from (Sorbent Technologies, Atlanta, GA, USA), according to published procedures [65,66]. TLC plates were spotted with sugar standards (sucrose, glucose and fructose reflect the major soluble sugars found in tobacco leaves), using a semi-automatic Linomat 5 sample applicator (Camag Scientific Inc., Wilmington, NC, USA). TLC plates were developed by using a mobile phase consisting of 65:20:15 acetonitrile:methanol:deionized water (v/v). The TLC plates were imaged by using autoradiography. After radiography, TLC plates were heat-treated at 200 °C for 15 min, to render the non-radioactive sugar standards visible under long-wave UV light [63,65], providing a means to correlate radioactive spots with leaf sugars. Radioactivity associated with the leaf sugars was summed and quantified by using ImageQuant TL 7.0 software. In the process, fixed-sized ROIs (regions of interest) were placed over each of the identified radioactive sugar spots where the software provided a numerical value for the spot intensity. The total [^11^C]sugar content was calculated by summing all the sugar ROIs and was related to the soluble extract ^11^C-activity by dividing this summed value by the total ^11^C-activity displayed on the sample lane of the TLC plate. All data were corrected for radioactive decay, using the following equation:

A_o_ = A_T_ × exp (λT)

where A_o_ is the calculated decay corrected radioactivity at T_o_ or EOB, A_T_ is the measured radioactivity at time T, λ is the decay constant equal to (ln2/t_½_) where t_½_ is the half-life for ^11^C equal to 20.4 min, and T is the elapse time from EOB to when the sample was counted.

### 3.6. Analysis of Plant Amino Acids

[^11^C & ^13^N]Amino acids were analyzed following published procedures [63,65] using pre-column OPA derivatization and quantified by gradient radio HPLC (Sonntek, Inc. Upper Saddle River, NJ, USA) using a Phenomenex Gemini 5 µm C18 (150 mm × 4.6 mm inner diameter) column heated to 30 °C and mobile phase system comprised of Solvent A (95:5 0.5 M sodium acetate: methanol) and Solvent B (70:30 methanol: 18 MΩ water) starting at 75:25 and switching to 25:75 within 30 min at a flow rate of 0.7 mL min^−1^. On-line fluorescence detection (340 nm excitation and 450 nm emission; Hitachi LaChrom Elite L-2485; Sonntek, Inc.) was used for quantification of the OPA-derivatized [^12^C]AAs and a NaI gamma radiation detector (Ortec-Ametek, Inc., Oak Ridge, TN, USA), enabling direct measurement of [^11^C]AAs. Data were acquired by using PeakSimple^TM^ (v4.88) chromatography software (SRI, Inc., Torrance, CA, USA). Retention times of eluting peaks were correlated to authentic standards for metabolite identification. Peaks were baseline integrated, providing a numerical value of the detector response. Radioactive metabolite peaks were quantified the same way, using the PeakSimple^TM^ (v4.88) software, corrected for radioactive decay and related back to a relative percent distribution of ^11^C activity found in the leaf tissue extract by applying a cross-calibration correction for differences in efficiency between the radiation flow detector and the gamma counter.

### 3.7. Synchrotron-Based X-Ray Microfluorescence

Synchrotron-based X-ray microfluorescence (µSXRF) images of leaf tissues were acquired from Beamline X27A of the National Synchrotron Light Source (NSLS) at Brookhaven National Laboratory (Upton, NY) [66]. Briefly, this beamline uses Kirkpatrick–Baez (KB) mirrors to produce a focused spot (7 by 14 µm) of hard X-rays with tunable energy achieved via Si(111) or Si(311) channel-cut monochromator crystals. For µSXRF imaging, the incident energy was fixed at 11 keV to excite all target elements simultaneously. Samples were oriented 45° to the incident beam, and rastered in the path of the beam by an XY stage, while X-ray fluorescence was detected by a Hitachi 4-element Vortex SDD detector positioned 90° to the incident beam. Elemental maps were collected from 2 mm diameter leaf tissue punches sampled from the treated and untreated sides of the same leaf. These samples were mounted on Kapton^TM^ foils for placement in the beamline. Scanning areas relied on step sizes of 50 µm for coarse mapping and 10 µm for fine mapping with a dwell time of 3 s. The fluorescence yields were normalized to the changes in intensity of the X-ray beam (I_0_) and the dwell time. Data acquisition and processing were performed by using IDL-based beamline software designed by CARS (U. Chicago, Consortium for Advanced Radiation Sources) and NSLS Beamline X26A (data analysis software available at http://www.bnl.gov/x26a/comp_download.shtml).

### 3.8. Statistical Analysis

Data were subjected to the Shapiro–Wilk Normality Test, to identify outliers so that all data groups reflected normal distributions. Temporal data were analyzed across a single JA treatment, using repeated measures ANOVA where the independent variable was time. Pair-wise comparisons were also made by using Student’s *t*-test, comparing the maximum fold-change in metabolites between 1 JA treatment and 2 JA treatments. Statistical significance was considered for a *p*-value < 0.05.

## 4. Conclusions

The complexity of plant defense responses that serve specialized chemistries in the production of protectant agents requires an abundant supply of energy and chemical substrates, primarily derived from primary metabolic processes. While these responses can come at a high cost to overall plant fitness, the consequences of not responding to attack can sometimes be more impactful. This can be relevant to plant–herbivore interactions, where, in some circumstances, tissue loss due to leaf-feeding insects can be so extensive that the plant does not survive the initial attack. However, these circumstances are rare. Sometimes, herbivore attacks come in waves. To increase long-standing resistance to waves of attack, plants have evolved an adaptable trait known as defense priming, where their response to subsequent attacks is accelerated and intensified. Here we presented evidence that the shikimate pathway and other ancillary primary metabolism associated with this pathway in *N. tabacum* are influenced by defense priming. Additionally, we have shown that these actions extend beyond plant utilization of C/N resources to include regulation of plant uptake of metal ions in times of stress. The extent to which these conclusions are generalizable to all higher plants will require further investigations. Furthermore, to better understand the influence and generalizability of defense priming on the cost and benefits to plant fitness and across plant species, we would need to expand the study to compare two herbivore-attacked plant systems—one with induced defenses and one without induced defenses.
